# Cultural diversity in early hearing detection and intervention: service provider perspectives

**DOI:** 10.1093/jdsade/enaf002

**Published:** 2025-03-12

**Authors:** Hayley Wong, Jane Sheehan, Valerie Sung, Stephanie Best, Greg Leigh

**Affiliations:** NextSense Institute, Sydney, Australia; Macquarie School of Education, Macquarie University, Sydney, Australia; Prevention Innovation, Population Health, Murdoch Children’s Research Institute, Melbourne, Australia; Prevention Innovation, Population Health, Murdoch Children’s Research Institute, Melbourne, Australia; Centre of Community Child Health, Royal Children’s Hospital Melbourne, Melbourne, Australia; Prevention Innovation, Population Health, Murdoch Children’s Research Institute, Melbourne, Australia; Centre of Community Child Health, Royal Children’s Hospital Melbourne, Melbourne, Australia; Department of Paediatrics, University of Melbourne, Melbourne, Australia; Australian Genomics, Murdoch Children's Research Institute, Melbourne, Australia; Melbourne School of Health Sciences, University of Melbourne, Melbourne, Australia; NextSense Institute, Sydney, Australia; Macquarie School of Education, Macquarie University, Sydney, Australia

## Abstract

This study investigated service providers’ perspectives on the barriers experienced by families from culturally and linguistically diverse backgrounds along the Early Hearing Detection and Intervention (EHDI) pathway in Victoria, Australia. Twelve service providers (i.e., hearing screening program staff and diagnostic audiologists) participated in semi-structured interviews. Data were analyzed using inductive content analysis. Service providers identified differences in service delivery, communication, and support needs between families from culturally and linguistically diverse backgrounds and those from majority cultural and linguistic (predominantly English-speaking) backgrounds. Perceived barriers included communication difficulties, lack of access to interpreters and translated written resources, cultural factors, and practical barriers to attending appointments. Clarifying the roles of service providers, providing access to resources to support communication, and requiring service providers to participate in cultural responsiveness training are suggested as strategies to improve services for families from culturally and linguistically diverse backgrounds. Findings from this study inform service provision throughout the EHDI pathway to improve care for families from culturally and linguistically diverse backgrounds.

Detection and diagnosis of hearing loss in newborns, and associated early intervention, have a significant impact on a range of developmental outcomes (Ching & Leigh, 2020; Wong et al., 2018; Yoshinaga-Itano, 2003). International best-practice guidelines for newborn hearing screening programs recommend that all eligible infants are screened for hearing loss by 1 month of age, with those referred to diagnostic audiology being assessed by 3 months of age (Department of Health and Aged Care, 2013; Joint Committee on Infant Hearing, 2019). In Australia, state and territory jurisdictions respond to a nationally defined target that 97% of infants with a positive screen result receive diagnostic assessment by 3 months corrected age (Australian Institute of Health and Welfare, 2013). Nevertheless, as demonstrated by Atherton et al. (2023), factors such as socioeconomic disadvantage, low parental education levels, and minority ethnicity can place some families at risk of nonadherence to these time frames. Indeed, in culturally diverse societies like Australia, there is a pressing need for healthcare providers to focus on the potential cultural and linguistic barriers encountered by families, to ensure equitable access to services such as hearing screening (Shukla et al., 2020).

Australia, like many countries, is a multicultural society where rapid immigration and the presence of significant populations of individuals of Indigenous descent have created a society where 22.8% of people use a language other than English (the majority language) at home, and more than 27.6% were born overseas (Australian Bureau of Statistics, 2022). Although, in international research literature, other terms might be used to describe people coming from such backgrounds, the term “cultural and linguistic diversity” is widely used in Australia to highlight the likely effects of both cultural *and* linguistic differences between these populations and the majority Anglo-European English-speaking population (Australian Bureau of Statistics, 1999; Pham et al., 2021).

Various researchers (Khatri & Assefa, 2022; Pham et al., 2021; White et al., 2019) have established that families from culturally and linguistically diverse backgrounds face significant challenges regarding their access to and use of health services. For example, lack of access to professional interpreting services (Hoang et al., 2009; Riggs et al., 2020), confusion about the roles of health professionals (Riggs et al., 2020), and unfamiliarity with disability services and the healthcare systems (Garg et al., 2017; Hoang et al., 2009; Zhou, 2016) have all been shown to inhibit access to essential healthcare information by individuals from culturally and linguistically diverse backgrounds. Passiveness in communication and a reluctance to be assertive in interactions with health service providers, who may be perceived as authority figures, have also been shown to account for difficulties experienced by families from culturally and linguistically diverse backgrounds (Liu et al., 2008; White et al., 2019). Further, family reactions of shame and/or guilt in response to a diagnosis of disability have been identified as barriers to seeking disability-specific support services for some parents from culturally and linguistically diverse backgrounds (Kvarme et al., 2017; Wynaden et al., 2005) because families may deny the possibility of a disability in their child (Woolfenden et al., 2015) or delay diagnostic testing in the hope that the condition will spontaneously resolve (The National Autistic Society, 2014). The stigma around disability can also lead to families disengaging from their community, as well as available support services, for fear of judgmental attitudes (The National Autistic Society).

Similar barriers have been reported in the limited research available regarding engagement with Early Hearing Detection and Intervention (EHDI) programs by families from culturally and linguistically diverse backgrounds. In an examination of diagnostic and therapy-related services to children with hearing loss in Canada, professionals reported cultural barriers related to the inability to attend sessions with professionals of the opposite gender, as well as instances of parents denying or hiding their child’s hearing loss, because disabilities were considered a source of shame in their culture (Grandpierre et al., 2019). Stigma and associated superstitious beliefs regarding the causes of disabilities were also identified in a study examining the attendance of families from various ethnic groups at follow-up diagnostic audiology appointments in Nigeria (Olusanya & Akinyemi, 2009). In that study, cultural barriers were a significant factor for the 39% of families that did not follow through to the diagnostic audiology stage of the EHDI pathway.

Communication difficulties—particularly regarding access to, and effective use of, interpreters—have been identified as a barrier to effective service delivery to families with limited ability to use the majority language when seeking to access services for their deaf or hard-of-hearing children (Grandpierre et al., 2019; Ng et al., 2022). In an Australian study (Ng et al., 2022), early intervention service providers reported that conveying information through interpreters often resulted in disconnection and difficulties in establishing relationships with families from linguistically diverse backgrounds. Additionally, the service providers reported providing less verbal and written information to families with limited English because of the restricted availability of resources in other languages. Any such barriers are likely to lead to delays or disruptions in diagnosis and intervention, which can have a significant impact on the language, social–emotional, and other developmental outcomes for children with hearing loss (Wong et al., 2018; Yoshinaga-Itano, 2003).

The studies considered give limited insight into the experiences of families from culturally and linguistically diverse backgrounds in the early stages of the EHDI pathway, a period that has been highlighted as a time of heightened stress and anxiety for many families (Fitzpatrick et al., 2007; Vohr et al., 2001). Given the importance of early identification of children with hearing loss for future outcomes, it is essential to better understand the barriers for families from culturally and linguistically diverse backgrounds during the early stages of the EHDI pathway (i.e., during screening and diagnostic audiology). This study aimed to identify and describe the experiences of service providers in the early stages of the EHDI pathway in the Australian state of Victoria, to understand the unique needs of working with families from culturally and linguistically diverse backgrounds.

## Context and study objectives

The Victorian Infant Hearing Screening Program (VIHSP) screens approximately 75,500 newborns each year (The Royal Children’s Hospital Melbourne, 2020). When an infant receives a “refer” result from screening for one or both ears, their parents/caregivers are referred to a diagnostic audiology clinic where audiologists perform a comprehensive hearing assessment to determine whether a temporary or permanent hearing loss is present. At the same time as referring to diagnostic audiology, VIHSP area managers connect families with the VIHSP Early Support Service. The service provides family-centered and child-focused support, using a case-facilitator model, to support optimal outcomes for infants with hearing loss by (a) guiding parents through the referral pathway and diagnostic audiology services; (b) facilitating parents’ understanding of the information provided; (c) attending to parents’ emotional needs; and (d) liaising with services and service providers to support the families’ timely engagement in services (Gillespie, 2013). Relative to a performance indicator of >90%, more than 99% of families are engaged with the Early Support Service within 3 days of notification (The Royal Children’s Hospital Melbourne, 2020). The VIHSP family support staff typically have a background in pediatric diagnostic audiology, early intervention or lived experience, or a combination of these characteristics.

This study aimed to explore the perspectives of service providers about working with families from a range of culturally and linguistically diverse backgrounds (including families from the Deaf community), in the context of the EHDI pathway in Victoria. The objectives were to:

1. identify service providers’ experiences of providing services for families from culturally and linguistically diverse backgrounds who are engaged in hearing screening, family support, and diagnostic audiology services;
2. describe differences between how service providers support families from culturally and linguistically diverse backgrounds and those from majority cultural and linguistic backgrounds; and
3. discern any potential areas of further support required for these families prior to and during confirmation of their infant’s hearing status.

## Method

This study adopted a qualitative approach to gain an in-depth understanding of the experiences of service providers when supporting families from culturally and linguistically diverse backgrounds. Semi-structured interviews were used to provide the flexibility to explore participants’ thoughts, feelings, and beliefs about a topic (DeJonckheere & Vaughn, 2019) and yield richer data than structured interviews (Al-Busaidi, 2008).

### Participants

Purposeful sampling was employed using the VIHSP clinical database to identify service providers along the EHDI pathway working at hospitals and centers with high proportions of families from culturally and linguistically diverse backgrounds. All service providers invited to participate were employed with VIHSP or a diagnostic audiology service in Victoria at the time of recruitment and had supported at least one family from a culturally and linguistically diverse background referred from VIHSP in the previous 12 months. Email invitations were sent to VIHSP Early Support Service family support staff, VIHSP area managers, and diagnostic audiologists, associated with six major hospitals and six audiology centers in Melbourne, Victoria. All participants gave informed consent prior to participating in the interviews. The study was approved by The Royal Children’s Hospital Human Research Ethics Committee (reference number 75949).

Twelve service providers from the EHDI pathway (all female) participated in semi-structured interviews at a location of their choosing. These included six hearing screening program staff (VIHSP family support staff and area managers—participants 1–6) and six diagnostic audiologists with experience in providing services for families from culturally and linguistically diverse backgrounds (participants 7–12).

### Procedure

We used a semi-structured interview format, including questions regarding (a) how the support needs for families from culturally and linguistically diverse backgrounds differ from those from the majority cultural and language population, (b) barriers to accessing hearing services for families from culturally and linguistically diverse backgrounds, and (c) how service providers can better support families from culturally and linguistically diverse backgrounds (Interview guide—Appendix A). Three participants took part in a group interview, while the remaining nine service providers completed individual interviews. Most interviews were conducted and recorded online via Zoom, with only one completed in the participant’s office. All interviews were conducted in spoken English.

### Data analysis

Interviews were fully transcribed from the recordings and subsequently cleaned and managed in NVivo 12 (QSR International Pty Ltd., 2018). Inductive content analysis was used to analyze the interview transcripts. With so little previous research being available to guide the investigation, this study was exploratory in nature and adopted an inductive approach (Elo & Kyngäs, 2008). This approach allowed for themes to be identified within the data and developed during the process of coding (Vears & Gillam, 2022). Content analysis also allowed for an exploration of participants’ personal experiences, to address the specific aims and objectives of this study (Erlingsson & Brysiewicz, 2017).

Three transcripts were independently coded by three members of the study team (H.W., J.S., S.B.). Coding was completed in two rounds, involving initial coding of high-level themes, followed by line-by-line coding, to further refine the themes with subthemes. Themes were compared and reviewed at regular team meetings until themes and sub-themes were agreed and accepted. One researcher (H.W.) then reviewed and completed coding for all transcripts. During the data analysis process, the study team discussed new themes and subthemes until no new responses to study aims were identified.

### Researcher positionality

Although not directly employed in any role within VIHSP, the primary researcher in this study (H.W.) is an “insider,” with background knowledge and professional associations with EHDI services (Hellawell, 2006). This insider knowledge allowed for a deeper level of understanding and interpretation of the data (Fleming, 2018). The primary researcher was also able to speak to participants at a familiar level during interviews (i.e., as a fellow service provider in the latter stages of the EHDI pathway). However, during the interviews, the primary researcher consciously kept to the role of interviewer and sought to maintain a neutral position. In the coding and analysis stage, selected transcripts were co-coded by a co-author who was an “outsider” to the field of pediatric hearing loss who could objectively code and discuss the data analysis from an “outsider” perspective.

## Results

Key findings from service providers’ responses centered around the three main topics of discussion. These were:

- common barriers encountered by families from culturally and linguistically diverse backgrounds when accessing or engaging in EHDI services (hereafter referred to as *barriers to services*).
- differences between the support needs of families from culturally and linguistically diverse backgrounds and those from majority cultural and linguistic backgrounds (hereafter referred to as *different service needs*).
- other areas of support that service providers suggested could improve accessibility and engagement of families from culturally and linguistically diverse backgrounds in EHDI services (hereafter referred to as *other suggested areas of support*).

### Barriers to services

Service providers identified multiple issues that they perceived to be barriers to culturally and linguistically diverse families engaging in the EHDI program. These barriers were categorized into three overarching themes: (1) communication between service providers and families, (2) cultural factors and external influences on families, and (3) practical barriers to engagement in services.

#### Communication between service providers and families

All service providers reported barriers to accessing interpreters for appointments and phone calls when needed. This was particularly challenging for the program staff, who described difficulties in accessing interpreters when the infant was settled or asleep and ready to be screened. Similarly, some audiologists described difficulties in booking interpreters for the two-hour diagnostic appointments:

[The interpreter] might just come at the start, disappear because they’ve been paid to go to another appointment and come back, so there’s often this juggling... you end up in a scenario where you’re like, okay the interpreter needs to go, how best will I work this? (participant 12).

When using interpreters to communicate with families, some service providers expressed uncertainty about the quality of interpreting. For example, participant 1 described situations where the parents and interpreters “talk for a very long time and then [the interpreters] just tell us one sentence,” resulting in limited communication and a reduction in the amount of support that could be provided via the interpreter. Most service providers reported that families often declined offers for interpreters, particularly when one parent can communicate in English. One service provider recognized a positive aspect to using family members as interpreters, stating that “sometimes it can feel very insensitive explaining results to a family through an interpreter. It’s easier through a family member” (participant 8). However, most described this as a difficult situation, particularly when the father was able to converse in English and declined an interpreter for the mother:

When the father’s there, his English is okay and he says, no mum doesn’t need an interpreter, but mum’s not really understanding... and so then you wonder how much information is getting back to mum. (participant 6).

Although one parent may be able to converse in English, service providers described the importance of offering an interpreter to the non-English speaking parent, to “[take] that burden and pressure off that English-speaking person, to be the bearer of that bad news” (participant 12). However, they reported being unsure about whether to insist on using an interpreter. In that regard, one provider shared an underlying reason that a family had declined an interpreter:

One family was saying if they got an interpreter, that they may be viewed as being seen that they weren’t integrating into our culture... their inability to understand or comprehend the English might be deemed as a bad thing. (participant 4).

Overall, even with interpreters present in appointments, service providers reported that misunderstandings still often occurred. Some language misunderstandings were not apparent until after the appointments, when information was fed back to service providers by the family support staff.

Most service providers stated that the number of written resources translated into languages other than English was small and substandard. This was especially problematic for the diagnostic audiologists, who send important information to families prior to their appointment, about the length of the appointment and how to prepare the infant. For the family support staff, the lack of translated resources resulted in loss to follow-up for some families from culturally and linguistically diverse backgrounds, who disengage from their services because “we can’t post them information to read privately” (participant 1). Other service providers opted to provide information in English to families from culturally and linguistically diverse backgrounds, in the hope that a family member or friend could assist in translating the information.

#### Cultural factors and external influences on families

Many service providers reported strong reactions of fear, shame, anger, and blame from parents from culturally and linguistically diverse backgrounds when told that their child may have a hearing loss:

I have had one mum tell me that she’s been blamed, that she did something wrong during her pregnancy and caused it... families have told me that they must have done something wrong in their previous life to have caused this in their child. (participant 1).

Consequently, service providers reported that some families asked to keep the results of the hearing test from their friends and family, or from other services, including the program staff, because they “don’t want the government to know” (participant 8). Service providers also gave examples of situations where parents sought information from other family members or medical professionals in their home country, resulting in confusion when given contradictory advice.

Additionally, some service providers observed that cultural beliefs can impact effective service delivery for some families. For example, one service provider described situations where she found gender differences in some cultural backgrounds as “very challenging” and commented that “the male can be very dominating” when attempting to communicate with the mother of the child (participant 5).

#### Practical barriers to engagement in services

Many service providers reported increased delays and difficulties in scheduling appointments with families from culturally and linguistically diverse backgrounds, compared to those from the majority culture who predominantly speak English. Service providers indicated that mothers were more likely to require support from the father for transport, communication in English, or care for other children. They noted that families from culturally and linguistically diverse backgrounds were more likely to cancel appointments at short notice or have difficulty rescheduling appointments around work or family commitments, resulting in delays in the EHDI pathway. One service provider suggested that barriers to finding care for other children was due to families’ financial situations:

A lot of [culturally and linguistically diverse families] can’t afford to book a babysitter for childcare, so that’s perhaps a financial constraint, so we often offer weekend appointments, so that the father who works normal business hours can stay home with siblings on the weekend or they can drive mum in and stay with the siblings. (participant 5).

### Different service needs

Service providers were asked to identify the main differences between supporting families from majority cultural and linguistic backgrounds and those from culturally and linguistically diverse backgrounds and to describe how they enabled the latter to engage in their services. Responses revealed three key themes: (1) differences in the provision of services for culturally and linguistically diverse families, (2) building connections with families from culturally and linguistically diverse backgrounds, and (3) support needs of families from culturally and linguistically diverse backgrounds.

#### Differences in provision of services for culturally and linguistically diverse families

Many service providers commented that providing services to families from culturally and linguistically diverse backgrounds required considerably more time than for their other families. They elaborated that often additional time was needed for information to be repeated, or questions to be asked, particularly when an interpreter was facilitating the conversation. However, one audiologist noted that additional time for appointments may be a function of a family’s relative level of socio-economic advantage/disadvantage:

Some people’s educational level can be quite low, and they actually will not understand the diagnosis... and engaging in multiple appointments can be reinventing the wheel each time... particularly when there’s interpreters involved, it’s a process. (participant 9).

Service providers noted that misunderstandings about services, hearing screens and tests, and the results of these tests, were more pronounced in families from culturally and linguistically diverse backgrounds. Service providers questioned how much families were understanding the pathway, indicating that families from culturally and linguistically diverse backgrounds were often just “going through the process” (participant 4). Additionally, families from culturally and linguistically diverse backgrounds were often confused about the different roles of service providers:

The other confusion sometimes, is with who they’re liaising with... so they think we’re the same people sometimes, and they’ll say “oh yeah, we just spoke to you the other day”... and then we realized that it wasn’t actually us that they spoke to. (participant 10).

Given their perceptions that families from culturally and linguistically diverse backgrounds were more likely to misunderstand information, many service providers spoke of using other services to support them in maintaining the engagement of these families, particularly when the parents were difficult to contact. Audiologists described working in partnership with the family support staff, highlighting the essential role they play in supporting families from culturally and linguistically diverse backgrounds along the EHDI pathway:

We’ll always email Early Support... VIHSP about what happened in the appointment, how the parents reacted, what went on, and we’ll often get feedback from there. (participant 8).

#### Building connections with families from culturally and linguistically diverse backgrounds

Service providers, particularly audiologists, reported that developing rapport with families from culturally and linguistically diverse backgrounds was more challenging than with families from majority cultural and linguistic backgrounds, especially when there was an interpreter involved. They described that the “emotional flow” and “natural empathy” can be lost in communication with families through an interpreter, resulting in appointments feeling “very matter of fact” (participant 12). Similarly, reading the emotional needs of parents was difficult when communication was not in English:

You can’t always judge when information’s coming back to you, what the family are telling the interpreter... It’s not actually from the words, it’s all the things around the words and that gets lost through an interpreter. (participant 8).

Service providers also reported that families from culturally and linguistically diverse backgrounds sometimes differed from families from the majority cultural and linguistic backgrounds in the questions they asked. They noted families from culturally and linguistically diverse backgrounds generally asked fewer questions, including questions of a clarifying nature:

Some cultures you do have that sense that... you listen to what the doctor says, and you don’t ask too many questions, so you might get fewer questions, or you might get more questions that ask for reassurance. (participant 9).

Contrastingly, other service providers noted that some families from culturally and linguistically diverse backgrounds took the “opportunity to get all the questions answered” when the interpreter was present, including “questions that might not be so related” (participant 7).

#### Support needs of families from culturally and linguistically diverse backgrounds

Most participants observed that families from culturally and linguistically diverse backgrounds were more likely to nominate the father as the primary contact for services because his English skills were generally better. They commented that this presented some additional challenges when obtaining consent from the mother, because some mothers would “always reflex to dad to answer all questions” (participant 5). One service provider questioned her cultural sensitivity in this situation:

Sometimes it’s hard to know... if the male is more dominant, and are we disrespecting him by talking to mum or are we disrespecting mum by talking to the father? (participant 4).

Having the father as the primary contact also presented problems when the father returned to work and couldn’t spend time speaking to service providers. One service provider observed that during this period, the mother needed more support because “she’s always on her own and doesn’t have anyone supporting her” (participant 1). Although service providers mentioned that some families from culturally and linguistically diverse backgrounds were more likely to have grandparents actively involved in appointments, some noted that these families “don’t generally talk about support from friends and the social network” (participant 3).

To support families that have fewer family and social networks, service providers reported offering to connect families with other people from a similar background or culture. Service providers also encouraged families to seek support from “somebody close by… that they trust… a health professional” (participant 8). Additionally, some service providers reported offering to speak directly to other family members or friends, to assist the family to understand the services and support available to them.

### Other suggested areas of support

Many service providers reported receiving very little feedback from families from culturally and linguistically diverse backgrounds about their services. Service providers expressed interest in knowing what families wanted from service providers and whether the information provided in the EHDI pathway was satisfactory, rather than “assuming we’re doing the right thing” (participant 4). Service providers also stated that further support could be offered to the second parent or extended family, to connect with family members “who have more influence over the family” (participant 8).

An area of improvement suggested by service providers related to their personal understandings about cultural influences on families. They described situations where they felt “it’s difficult to know what might offend and what won’t” (participant 4) and wanted “an understanding about where families have come from, what their background is” (participant 8). Service providers expressed a desire for more cultural training and ways to obtain further information and resources about specific cultures. One service provider suggested that using community links, such as a community liaison officer, may assist in engaging families.

Finally, service providers highlighted that little is known about why certain families from culturally and linguistically diverse backgrounds disengage from EHDI programs, particularly when they are not able to establish contact with families. However, the program staff reported that a recent introduction of translated text messages through an approved online SMS platform gave a promising opportunity to engage these families:

We’ve sent out text messages in English, and parents have not responded and not turned up for those appointments... we’ve then started to use [the SMS platform], we will send the English message... and then we will also send the same message in whichever language... and parents have replied, and they’ve attended. (participant 3).

## Discussion

This study explored service providers’ experiences of working with families from culturally and linguistically diverse backgrounds in the early stages of the EHDI pathway, including how service providers support these families and potential areas of further support. Interviews with service providers generated insights into the specific needs of families from culturally and linguistically diverse backgrounds, to identify potential ways to improve access to and engagement in EHDI programs for these families.

### Language and communication needs of families from culturally and linguistically diverse backgrounds

Language and communication difficulties were commonly reported barriers by service providers. Specifically, they expressed concerns when parents opted for family members to interpret during appointments, echoing findings from previous studies (Riggs et al., 2020; White et al., 2019). Although one service provider observed that family members as interpreters sometimes facilitated difficult conversations, the results here and the conclusions of previous research serve to confirm the importance of using professional, qualified interpreters to ensure the quality, safety, and effectiveness of health care (Riggs et al., 2020; Torresdey et al., 2024). Additionally, it is noteworthy that relevant professional guidelines state that family members should remain in a support and advocacy role for clients, separate from the role of a qualified interpreter (Department of Health and Human Services, 2017). To reduce risks of miscommunication leading to poorer quality of care, the cumulative evidence suggests that service providers should make every reasonable effort to use a qualified interpreter for the provision of services when required.

Service delivery was also hindered when families declined an offer of an interpreter during appointments. Families with a basic level of English were not necessarily aware of the limited information they were receiving. Notably, one service provider described a family declining an interpreter because they were ashamed that they needed one. This finding has not previously been reported in the literature but does extend previous studies that found patients from culturally and linguistically diverse backgrounds felt guilty or uncomfortable to ask for an interpreter, for fear of being a burden on the health system (White et al., 2019).

Some audiologists also identified instances of language discordance, when they were unaware that families had misunderstood information until after appointments, through feedback from the family support staff. Reasons for language discordance may be related to the quality of interpreting (i.e., through a qualified interpreter or unqualified family member), limited time during appointments, or families adopting a passive role during discussions. This finding highlights the importance of the role of the family support staff, in collaborating with services to track and support families from culturally and linguistically diverse backgrounds.

Previous research has shown that using written information can support parents’ understanding of the pathway and clarify misconceptions (Scarinci et al., 2018; Scheepers et al., 2014). However, service providers here reported that written resources were currently inadequate and could not cover all the languages and information they required. The reasons for this are unclear; however, they are likely related to limitations in funding and the availability of human resources to translate and verify written information in a wider range of languages (Parliament of Victoria, 2020). Regardless, some service providers reported providing resources in English, hoping that there would be a family member or friend to translate it. Although the use of English-based resources, supported by pictures or diagrams, may increase accessibility for families from culturally and linguistically diverse backgrounds (Department of Health and Aged Care, 2013; Uebergang et al., 2021), access to written resources in their home language remains the preference for supporting these families in health services (Riggs et al., 2020).

### Social–emotional needs

Service providers did not identify any differences between the level of concern expressed about the EHDI process by families from culturally and linguistically diverse backgrounds and those from majority cultural and linguistic backgrounds. However, they acknowledged that it was harder to gauge the emotions of families from culturally and linguistically diverse backgrounds, particularly when communicating through an interpreter. This has been identified in other studies examining the use of health services by people from culturally and linguistically diverse backgrounds, leading to the suggestion that longer appointment times should be made available to these families, to allow parents more time to express their concerns and have their questions answered by service providers (Torresdey et al., 2024).

In this study, service providers observed a distinct difference between families from culturally and linguistically diverse backgrounds and those from majority cultural and linguistic backgrounds regarding their ability to access extended family and other community support. This is consistent with other research showing that families from culturally and linguistically diverse backgrounds are at greater risk of being isolated and are unaware of how to seek support from the local community (Hoang et al., 2009). Interestingly, some service providers observed that families with limited social support were more engaged in their services. Nevertheless, they suggested opportunities to connect with service providers and other families who speak the same language would be beneficial, supporting previous research conclusions that speaking to others of the same background can reduce feelings of isolation (Ng et al., 2022). However, speaking to other family members and community members can influence parents’ decisions relating to their child’s disability and negatively impact families’ engagement with services (Woolfenden et al., 2015). Therefore, service providers suggested that offering to speak to other family members or close friends may address some of these challenges and educate the family and their supports about the importance of the EHDI pathway.

### Cultural impacts

Service providers in this study suggested that shame and stigma associated with disabilities in certain cultures were a barrier to engaging in EHDI services for some families. Previous studies have reported similar observations, noting that families from certain cultural backgrounds may be reluctant to talk about their child’s potential hearing loss because it is considered a source of shame (Grandpierre et al., 2019; Olusanya & Akinyemi, 2009). Service providers also expressed uncertainty regarding other cultural beliefs, particularly relating to gender role differences in some cultures, echoing similar findings that limited understanding of cultural differences presented challenges in their service provision (Ng et al., 2022). Service providers highlighted that their own knowledge of how cultural values and beliefs may affect families from some culturally and linguistically diverse backgrounds was limited, and they suggested that further training in this area would be beneficial.

Service providers also reported encountering challenges when families indicated that they had received contradictory information or opinions from family or medical professionals in their home country. This is consistent with findings reported by Woolfenden et al. (2015). This may have led to delays in confirmation of hearing loss for some families that delayed attending follow-up appointments until they confirmed that further action was required. It would appear important, therefore, that service providers develop a trusting, positive relationship with families (Garg et al., 2017), to encourage families to ask questions, clarify information, and comfortably follow the advice given by service providers. Furthermore, this highlights the need for families to have access to high-quality information in their home language, through translated resources, or to be directed to appropriate resources from hearing support services based in their home country, that will support their understanding of the EHDI pathway.

### Ease of access to services

Service providers reported that lack of interpreter support was a substantial barrier to engagement in services, severely limiting the communication between the parties. This finding aligns with previous research that identified the unavailability of interpreters, particularly those for uncommon languages or dialects, as a cause of lower quality and effectiveness of health care (Riggs et al., 2020). This collective evidence supports the importance of ensuring that families from culturally and linguistically diverse backgrounds have access to interpreters for all stages of the EHDI pathway, to ensure a clear understanding of the information provided and to stress the importance of timely completion of the pathway for their infant. The unavailability of interpreters may continue to be an ongoing barrier in the future, due to limited resources and logistical challenges; therefore, services may need to explore other options to support communication in families’ home languages, such as using translated written resources or online text translations, with verification from professionals to ensure accuracy.

Service providers also reported that families encountered other barriers to accessibility of services, such as difficulty arranging transport, caring for other children, and attending appointments around work and family commitments. Although previous studies indicate that these logistical barriers are also present in families from majority cultural and linguistic backgrounds (Laugen, 2013), parenting responsibilities and transport barriers have been found to be key barriers to culturally and linguistically diverse families’ participation in other health programs (Cyril et al., 2017). Service providers in this study highlighted that mothers from culturally and linguistically diverse backgrounds are more likely to be reliant on the father for transport and communication, emphasizing the need for flexibility of services in offering appointments around work and family commitments.

### Clarifying the EHDI pathway and services for families from culturally and linguistically diverse backgrounds

In this study, service providers reported that misunderstandings and lack of knowledge were common occurrences for many families from culturally and linguistically diverse backgrounds as they negotiated the EHDI pathway. This is consistent with previous research in this area (Russ et al., 2004; Scheepers et al., 2014). Furthermore, service providers in this study reported that uncertainty regarding the roles of service providers was more pronounced in families from culturally and linguistically diverse backgrounds. It was reported that many families were not aware of who the family support staff were and were underutilizing this vital service, leading to unanswered questions, ongoing concerns, and further misunderstandings relating to the pathway. This may be due to language barriers or unfamiliarity with the healthcare system (Riggs et al., 2020), suggesting the importance of service providers proactively supporting families from culturally and linguistically diverse backgrounds, to ensure multiple opportunities to inform and clarify information about the EHDI pathway.

Previous studies examining the EHDI pathway have noted the importance of having coordinated services and continuously monitoring families through each stage of the process, to reduce the risk of delays and disengagement (Danhauer & Johnson, 2006; Scarinci et al., 2018). Service providers in this study emphasized the importance of family support (i.e., in this context, the VIHSP Early Support Service) for families from culturally and linguistically diverse backgrounds. They emphasized the need for significantly more time and effort to be invested to ensure that families remain engaged in the EHDI pathway. Additionally, the program staff noted that they proactively contact families from culturally and linguistically diverse backgrounds, suggesting that this is necessary to support parents who may be unaware of, or unable to, access avenues for information or support. Therefore, findings from this study support research from other successful universal newborn hearing screening programs, in which collaboration across services and effective follow up have been shown to be pivotal for families who are at higher risk of disengagement (Liu et al., 2008).

### Implications for service delivery

Findings from this study highlight the importance of family support staff being embedded within EHDI programs, particularly in supporting families from culturally and linguistically diverse backgrounds. It should be acknowledged, however, that the evidence presented here suggests that, without additional efforts, these families are less likely to utilize this support. To give families from culturally and linguistically diverse backgrounds the opportunity to seek such support when needed, EHDI programs should provide clear information about the roles of service providers and the support that can be offered to families in their home language. Additionally, service providers need to proactively engage and maintain contact with families from culturally and linguistically diverse backgrounds, before, during, and after appointments, in a collaborative and coordinated approach, to reduce the risk of delays or disengagement with services, due to a lack of information or understanding of services.

Hospitals, audiology clinics, and family support services should ensure adequate provision of interpreters. Although engaging interpreters at the right time is not always logistically possible, interpreters should always be offered to families as a first step in service provision. Training service providers in the effective use of interpreters may also be beneficial when supporting families with limited English. This training could include strategies to (a) avoid jargon and confusing terminology, (b) determine the need for an interpreter with families with a basic level of English, and (c) encourage families to recognize the importance of having an interpreter present (i.e., to ensure families understand the importance of engaging in the EHDI pathway for their infant).

Services should continue to invest funding and resources into producing high-quality, current information in a range of languages and dialects to ensure equity of access to resources for families from culturally and linguistically diverse backgrounds with limited English skills. These resources should be easily accessible in multiple formats, including audio-visual material. Additionally, assisting families to connect with support groups may give some the opportunity to engage with other families from a similar cultural background, to share questions, concerns, and experiences. Online text translations may offer an additional resource for service providers to connect with families who may be difficult to contact and are at risk of disengaging from services. However, caution must be taken to ensure the accuracy of translated information.

Finally, service providers suggested a need for more cultural responsiveness training, to improve their understanding of the cultural backgrounds of families and their beliefs. Other studies have suggested that opportunities to work alongside community liaison officers, community leaders, or bilingual–bicultural service providers, could also encourage better engagement and support for families from culturally and linguistically diverse backgrounds (Woolfenden et al., 2015), which may also assist them in accessing appropriate information and support from their country of origin (i.e., via the Internet). Building on the cultural responsiveness of service providers may also help families to build rapport and trust in service providers. This can include training and the introduction of competency standards that promote respect and sensitivity to different cultures within family-centered care (Migrant and Refugee Women’s Health Partnership, 2019). Such measures may, in turn, reduce the risk of delays in or disengagement from the pathway. By developing the cultural responsiveness of service providers in the EHDI pathway, services can endeavor to provide families from culturally and linguistically diverse backgrounds with the same quality and quantity of information that is given to families whose primary language is English (Yoshinaga-Itano, 2014).

### Limitations

The use of purposeful sampling in this study ensured the inclusion of service providers who were most likely to have extensive experience in supporting families from culturally and linguistically diverse backgrounds. Therefore, these providers came from just one metropolitan region and the study did not examine service provision for families in rural or remote regions. The perspectives of service providers interviewed in this study referred to aspects specific to VIHSP and the diagnostic pathway in Victoria and may not be pertinent to other universal newborn hearing screening programs around Australia or internationally.

Member checks were not employed in this study. Revision of transcripts or summary of results by participants in future studies may provide opportunities for feedback and refinement of key ideas and meaning for rigor.

The focus of this study was to describe service providers’ perspectives on the barriers experienced by families from culturally and linguistically diverse backgrounds along the early stages of the EHDI pathway. Exploring the engagement of families from culturally and linguistically diverse backgrounds in longer-term programs may present similar issues; however, it could also raise additional barriers that have not been identified in this study. Therefore, further research exploring the whole EHDI pathway for families from culturally and linguistically diverse backgrounds is recommended for future investigations. In a related study, we explored culturally and linguistically diverse families’ perspectives of the early stages of the EHDI pathway, which will be reported elsewhere.

## Conclusion

This study starts to fill the gap in understanding the barriers that families from culturally and linguistically diverse backgrounds experience when negotiating the early stages of EHDI programs. In doing so, it has identified some strengths in how service providers support these families and recommends further strategies to reduce the risk of delays or disengagement of these families from the EHDI pathway. Further research into the experiences of families from culturally and linguistically diverse backgrounds across the entire EHDI pathway is essential to build more equitable services for all families and further reduce the gaps in knowledge regarding families from culturally and linguistically diverse backgrounds in health-related research.

## Appendix

**A:** Interview guide

**Key questions**	**Example prompt questions**
**Introduction:** Can you please introduce yourself and tell me about your role in supporting families through the hearing screening pathway?	What is your main role? How long have you been working at this organization/service?
**Engaging in services:** Compared to families from English-only-speaking backgrounds,... How do families from culturally and linguistically diverse backgrounds differ from other families in their support needs when accessing your services? What do you feel are the barriers to culturally and linguistically diverse families accessing your services? Possible answers/themes—refer to prompt questions: ▪ Emotional support/family ▪ Understanding of services ▪ Cultural influences/family factors ▪ Language factors ▪ Other factors What factors made it easier for culturally and linguistically diverse families to access your services? Possible answers/themes—refer to prompt questions: ▪ Emotional support/family ▪ Understanding of services (i.e., resources) ▪ Language factors ▪ Other factors	**Emotional support for parents/family:** Which member of the family do you have the most contact with? How does this impact your services? Usually, how involved are the other family members (in particular the second parent) in your services? What issues/questions raised by parents might indicate that they require further support? In your experience, what sources of information or support do culturally and linguistically diverse parents use?
	**Understanding of services:** What questions do you hear from parents from culturally and linguistically diverse backgrounds? What misunderstandings do you encounter about your services or the hearing screening pathway? Could you tell me about any resources that you use that are specifically for families from culturally and linguistically diverse backgrounds? How accessible are the resources that you use, for families from culturally and linguistically diverse backgrounds?
	**Cultural influences/family factors:** How have families’ cultural or religious beliefs impacted your services? How have location or transport factors impacted on culturally and linguistically diverse families’ access to your services? In your experience, how often have parents raised concerns about finances or immigration status in discussions with you? How are other family members, such as grandparents, involved in your services? How often have you experienced culturally and linguistically diverse parents’ reluctance to tell other family members about their child’s possible hearing loss? What impact does this have on your services? How often has lack of extended family support impacted on culturally and linguistically diverse families’ accessing your services?
	**Language factors:** Could you describe any situations where language has been a significant barrier to families’ access to your services? What do you do to resolve language misunderstandings when speaking to families? In your experience, what translated resources are helpful for culturally and linguistically diverse families? Are there any others that you think could be helpful?
	**Other factors:** In your experience, what other factors have influenced families in engaging in your services, talking to you, returning phone calls, etc.? In your experience, what factors delay a culturally and linguistically diverse family from confirming the hearing status of their child? What impact has Covid-19 had on culturally and linguistically diverse families’ access to or participation in your services?
**Future supports or improvements** What would help you provide better services specifically for families from culturally and linguistically diverse backgrounds?	What feedback do you get from parents about your services? Following these interviews, we will be doing interviews with families from culturally and linguistically diverse backgrounds. What questions would you like to ask these families about their experiences in accessing your services? What change to your services do you feel would make the most impact for supporting families from culturally and linguistically diverse backgrounds? Do you have anything else that you would like to add that we haven’t spoken about?

**B:** List of codes and examples

**Theme**	**Sub-theme**	**Example**
Communication and connections	Asking questions	“Because of their… language barrier, they want to ask you all the questions while you’re there with the interpreter, so it’s like their opportunity to get all the questions answered and even ask other questions that might not be so related. Um so they really, yeah, jump on that opportunity” (participant 7)
	Language misunderstandings	“Understanding of the other subtleties is probably a lot more challenging for a lot of [culturally and linguistically diverse] families, because we use a lot more words, ... I certainly spend a large chunk of appointments talking, when things aren’t all fine, for them, which is likely a lot harder when we can’t easily communicate for them” (participant 11)
	Rapport with family	“I think sometimes it is hard to read exactly how the parents are feeling, when you’ve got that sort of, triangle if you like, you know, the interpreter, you’re not exactly sure what the parents are saying to each other” (participant 10)
Cultural factors and influences	Cultural beliefs—gender	“She’s always reflexing to dad to answer all questions. And I get the sense that mum does understand, but it’s always been the case that, no no, talk to my husband” (participant 5)
	Understanding cultural influences	“We have to have an understanding about where families have come from, what their background is, so there’s all sorts of cultural factors” (participant 8)
	Stigma or shame	“Sometimes there’s a mistrust of government or authority, depending on where they’ve come from… I’ve had families when I’ve diagnosed a hearing loss, say please don’t tell anybody... I don’t want the government to know” (participant 8)
Differences in service provision	More support needed	“We just try to link them in with everyone that we can, without overwhelming them, that that’s the difficulty because, when that does happen, they, they’re often wanting to retreat... just try to work out what it is they need, to get them the next small step along the way” (participant 9)
	More time needed	“Our role is providing emotional and informational support to the family so I’m trying to get through, that informational support all takes quite a bit longer” (Participant 2)
Engagement in services	Practical barriers	“I’ve actually done a few [appointments] in the carpark because parents can’t have the other kids looked after so… for those parents that can’t come in on their own” (participant 6)
	Comparison to home country	“They explain back in their country things are done a bit differently so they wonder whether we can, fix things, ..., they’re not quite understanding what the problem is” (participant 7)
	Disengaging, delaying, or declining services	“We will have some families who don’t engage at all, particularly some cultures, .. . they don’t want the audiologist to tell us what the diagnosis is. .. they decline our support and that’s usually [culturally and linguistically diverse] families” (participant 1)
	Follow-up and tracking	“The other thing we can sometimes try is maternal and child health nurses can help us out, so if we’re not able to make any contact whatsoever with families,... if the family is not attending appointments or sort of following the pathway, then the maternal and child health nurse, if she is engaged with them, can be really helpful” (participant 2)
	Obtaining feedback	“We honestly don’t get enough feedback,... because we often send kids off and we don’t know what happens next, it’s not just Hearing Australia, just in general we don’t necessarily hear a lot about how kids are going” (participant 9)
	Understanding services and service providers	“We just slow it right back, go back right to the beginning, and I’ll go through the whole process again... that just kind of alludes to the fact that they don’t quite get it... they’ll go through the process, but are they understanding each step of it? Not always entirely sure that they are” (participant 4)
Interpreters and interpreting	Access to interpreters	“If that parent runs late, if the baby’s unsettled, then the booking of that interpreter goes right out the window, and often we’re making appointments for interpreters, and then the family arrive, and… the interpreters might not be available, so then we’re sort of left, kind of just hanging” (participant 4)
	Declining interpreters, using informal interpreters	“family members at times, when they’re being the translator, sometimes you wonder how much they’re actually telling the other person, ‘cause you say a whole paragraph and they’ve just said one sentence” (participant 7)
	Quality of interpreting	“And there is always the challenge of using interpreters, that, they talk for a very long time and then they just tell us one sentence, and…, the amount of support we can provide. . . is limited” (participant 1)
Written information	In English	“We certainly have documents that they can take home when there’s been a diagnosis, and they’re largely in English… some of them where the family’s been here for a while, there’s a little bit of English, and you’re sure there are other people in their circle who do speak quite good English, they’ll get someone to read it to them, so I’ll happily give an English version, to some non-English speakers” (participant 9)
	In other languages	“Certainly the brochures are a huge support...when the screeners are out on the ward.... If there is no English by mum, then at least the screener can begin with, by leaving a LOTE brochure. And then following through with the interpreter service” (participant 3)

## References

[ref1] Al-Busaidi, Z. Q. (2008). Qualitative research and its uses in health care. Sultan Qaboos University Medical Journal, 8(1), 11–19. 21654952 PMC3087733

[ref2] Atherton, K. M., Poupore, N. S., Clemmens, C. S., Nietert, P. J., & Pecha, P. P. (2023). Sociodemographic factors affecting loss to follow-up after newborn hearing screening: A systematic review and meta-analysis. Otolaryngology-Head and Neck Surgery, 168(6), 1289–1300. 10.1002/ohn.221 36939626 PMC10773460

[ref3] Australian Bureau of Statistics. (1999). Standards for statistics on cultural and language diversity. Canberra: ABS.

[ref4] Australian Bureau of Statistics. (2022, September 20). Cultural diversity of Australia. ABS. https://www.abs.gov.au/articles/cultural-diversity-australia.

[ref5] Australian Institute of Health and Welfare. (2013). National performance indicators to support neonatal hearing screening in Australia. Canberra: AIHW.

[ref6] Ching, T. Y. C., & Leigh, G. (2020). Considering the impact of universal newbron hearing screening and early intervention on language outcomes for children with congenital hearing loss. Hearing, Balance and Communication, 18(4), 215–224. 10.1080/21695717.2020.1846923 34249584 PMC8270008

[ref7] Cyril, S., Nicholson, J. M., Agho, K., Polonsky, M., & Renzaho, A. M. (2017). Barriers and facilitators to childhood obesity prevention among culturally and linguistically diverse (CALD) communities in Victoria, Australia. Australian and New Zealand Journal of Public Health, 41(3), 287–293. 10.1111/1753-6405.12648 28245512

[ref8] Danhauer, J. L., & Johnson, C. E. (2006). A case study of an emerging community-based early hearing detection and intervention program: Part I. Parents' compliance. American Journal of Audiology, 15(1), 25–32. 10.1044/1059-0889(2006/004) 16803789

[ref9] DeJonckheere, M., & Vaughn, L. M. (2019). Semistructured interviewing in primary care research: A balance of relationship and rigour. Family Medicine and Community Health, 7(2), e000057. 10.1136/fmch-2018-000057 32148704 PMC6910737

[ref10] Department of Health and Aged Care. (2013). National framework for neonatal hearing screening. Author. https://www1.health.gov.au/internet/main/publishing.nsf/Content/F2CA5966778F5842CA257C05001BFE12/$File/Neonatal%20hearing%20screening%20framework%20-%20August%202013.pdf.

[ref11] Department of Health and Human Services. (2017). Language services policy. The Victorian Government. https://www.dhhs.vic.gov.au/publications/language-services-policy-and-guidelines.

[ref12] Elo, S., & Kyngäs, H. (2008). The qualitative content analysis process. Journal of Advanced Nursing, 62(1), 107–115. 10.1111/j.1365-2648.2007.04569.x 18352969

[ref13] Erlingsson, C., & Brysiewicz, P. (2017). A hands-on guide to doing content analysis. African Journal of Emergency Medicine, 7(3), 93–99. 10.1016/j.afjem.2017.08.001 30456117 PMC6234169

[ref14] Fitzpatrick, E., Graham, I. D., Durieux-Smith, A., Angus, D., & Coyle, D. (2007). Parents' perspectives on the impact of the early diagnosis of childhood hearing loss. International Journal of Audiology, 46(2), 97–106. 10.1080/14992020600977770 17365061

[ref15] Fleming, J. (2018). Recognizing and resolving the challenges of being an insider researcher in work-integrated learning. International Journal of Work-Integrated Learning, 19(3), 311.

[ref16] Garg, P., Ha, M. T., Eastwood, J., Harvey, S., Woolfenden, S., Murphy, E., Dissanayake, C., Jalaludin, B., Williams, K., McKenzie, A., Einfeld, S., Silove, N., Short, K., & Eapen, V. (2017). Explaining culturally and linguistically diverse (CALD) parents' access of healthcare services for developmental surveillance and anticipatory guidance: Qualitative findings from the 'Watch me Grow' study. BMC Health Services Research, 17(1), 228–228. 10.1186/s12913-017-2143-1 28330490 PMC5361826

[ref17] Gillespie, J. (2013, May 18). *VIHSP early support service* [PowerPoint slides]. Australasian Newborn Hearing Screening Committee. https://www.newbornhearingscreening.com.au/wp-content/uploads/2012/05/Sat-3B-1330-J-Gillespie.pdf.

[ref18] Grandpierre, V., Nassrallah, F., Potter, B. K., Fitzpatrick, E. M., Thomas, R., Taylor, J., & Sikora, L. (2019). Examining cultural competence in pediatric hearing loss services: A survey. Deafness and Education International, 21(4), 174–194. 10.1080/14643154.2019.1589075

[ref19] Hellawell, D. (2006). Inside-out: Analysis of the insider-outsider concept as a heuristic device to develop reflexivity in students doing qualitative research. Teaching in Higher Education, 11(4), 483–494. 10.1080/13562510600874292

[ref20] Hoang, H. T., Le, Q., & Kilpatrick, S. (2009). Having a baby in the new land: A qualitative exploration of the experiences of Asian migrants in rural Tasmania, Australia. Rural and Remote Health, 9(1), 1084–1084. 10.22605/RRH1084 19243226

[ref21] Joint Committee on Infant Hearing. (2019). Year 2019 position statement: Principles and guidelines for early hearing detection and intervention programs. Journal of Early Hearing Detection and Intervention, 4(2), 1–44. 10.15142/fptk-b748 10943021

[ref22] Khatri, R. B., & Assefa, Y. (2022). Access to health services among culturally and linguistically diverse populations in the Australian universal health care system: Issues and challenges. BMC Public Health, 22, 880. 10.1186/s12889-022-13256-z 35505307 PMC9063872

[ref23] Kvarme, L. G., Alebertini Früh, E., & Lidèn, H. (2017). How do immigrant parents of children with complex health needs manage to cope in their daily lives? Child & Family Social Work, 22(4), 1399–1406. 10.1111/cfs.12358

[ref24] Laugen, N. J. (2013). Providing information to families in newborn hearing screening follow-up: Professional challenges. Seminars in Hearing, 34(1), 011–018. 10.1055/s-0032-1333147

[ref25] Liu, C.-L., Farrell, J., MacNeil, J. R., Stone, S., & Barfield, W. (2008). Evaluating loss to follow-up in newborn hearing screening in Massachusetts. Pediatrics (Evanston), 121(2), e335–e343. 10.1542/peds.2006-3540 18187812

[ref26] Migrant and Refugee Women’s Health Partnership. (2019). Culturally responsive clinical practice: Working with people from migrant and refugee backgrounds. Author. https://culturaldiversityhealth.org.au/wp-content/uploads/2019/02/Culturally-responsive-clinical-practice-Working-with-people-from-migrant-and-refugee-backgrounds-Jan2019.pdf.

[ref27] Ng, Z. Y., Waite, M., Ekberg, K., & Hickson, L. (2022). Clinicians' and managers' views and experiences of audiology and speech-language pathology service provision for culturally and linguistically diverse families of young children with hearing loss. Journal of Speech, Language, and Hearing Research, 65(7), 2691–2708. 10.1044/2022_JSLHR-21-00378 35738009

[ref28] Olusanya, B. O., & Akinyemi, O. O. (2009). Community-based infant hearing screening in a developing country: Parental uptake of follow-up services. BMC Public Health, 9(1), 66–66. 10.1186/1471-2458-9-66 19236718 PMC2656536

[ref29] Parliament of Victoria. (2020). Inquiry into early childhood engagement of culturally and linguistically diverse communities. Author. https://www.parliament.vic.gov.au/lsic-la/inquiries/article/4236.

[ref30] Pham, T. T. L., Berecki-Gisolf, J., Clapperton, A., O'Brien, K. S., Liu, S., & Gibson, K. (2021). Definitions of culturally and linguistically diverse (CALD): A literature review of epidemiological research in Australia. International Journal of Environmental Research and Public Health, 18(2), 737. 10.3390/ijerph18020737 33467144 PMC7830035

[ref31] QSR International Pty Ltd (2018). *NVivo* (Vol. 12). https://www.qsrinternational.com/nvivo-qualitative-data-analysis-software/home?_ga=2.87281688.1273848862.1668328728-980144146.1652701443.

[ref32] Riggs, E., Yelland, J., Szwarc, J., Duell-Piening, P., Wahidi, S., Fouladi, F., Casey, S., Chesters, D., & Brown, S. (2020). Afghan families and health professionals’ access to health information during and after pregnancy. Women and Birth : Journal of the Australian College of Midwives, 33(3), e209–e215. 10.1016/j.wombi.2019.04.008 31097412

[ref33] Russ, S. A., Kuo, A. A., Poulakis, Z., Barker, M., Rickards, F., Saunders, K., Jarman, F. C., Wake, M., & Oberklaid, F. (2004). Qualitative analysis of parents’ experience with early detection of hearing loss. Archives of Disease in Childhood, 89(4), 353–358. 10.1136/adc.2002.024125 15033847 PMC1719881

[ref34] Scarinci, N., Erbasi, E., Moore, E., Ching, T. Y. C., & Marnane, V. (2018). The parents' perspective of the early diagnostic period of their child with hearing loss: Information and support. International Journal of Audiology, 57(sup2), S3–S14. 10.1080/14992027.2017.1301683 PMC5681436 28332410

[ref35] Scheepers, L. J., Swanepoel, D. W., & Roux, T. l. (2014). Why parents refuse newborn hearing screening and default on follow-up rescreening—A south African perspective. International Journal of Pediatric Otorhinolaryngology, 78(4), 652–658. 10.1016/j.ijporl.2014.01.026 24560238

[ref36] Shukla, N., Pradhan, B., Dikshit, A., Chakraborty, S., & Alamri, A. M. (2020). A review of models used for investigating barriers to healthcare access in Australia. International Journal of Environmental Research and Public Health, 17(11), 4087. 10.3390/ijerph17114087 32521710 PMC7312585

[ref37] The National Autistic Society (2014). Diverse perspectives: The challenges for families affected by autism from black, Asian and minority ethnic communities. Author. https://s3.chorus-mk.thirdlight.com/file/1573224908/63849355948/width=-1/height=-1/format=-1/fit=scale/t=445333/e=never/k=7c17beeb/Diverse-perspectives-report.pdf.

[ref38] The Royal Children’s Hospital Melbourne (2020). News and updates - Victorian newborn hearing screening pathway. Author. https://www.rch.org.au/vihsp/news_and_updates/.

[ref39] Torresdey, P., Chen, J., & Rodriguez, H. P. (2024). Patient time spent with professional medical interpreters and the care experiences of patients with limited English proficiency. Journal of Primary Care & Community Health, 15, 21501319241264168. 10.1177/21501319241264168 PMC11265237 38912573

[ref40] Uebergang, E., Best, S., de Silva, M. G., & Finlay, K. (2021). Understanding genomic health information: How to meet the needs of the culturally and linguistically diverse community—A mixed methods study. Journal of Community Genetics, 12(4), 549–557. 10.1007/s12687-021-00537-0 34185265 PMC8554909

[ref41] Vears, D. F., & Gillam, L. (2022). Inductive content analysis: A guide for beginning qualitative researchers. Focus on Health Professional Education, 23(1), 111–127. 10.11157/fohpe.v23i1.544

[ref42] Vohr, B. R., Letourneau, K. S., & McDermott, C. (2001). Maternal worry about neonatal hearing screening. Journal of Perinatology, 21(1), 15–20. 10.1038/sj.jp.7200475 11268862

[ref43] White, J., Plompen, T., Tao, L., Micallef, E., & Haines, T. (2019). What is needed in culturally competent healthcare systems? A qualitative exploration of culturally diverse patients and professional interpreters in an Australian healthcare setting. BMC Public Health, 19(1), 1096–1096. 10.1186/s12889-019-7378-9 31409317 PMC6693250

[ref44] Wong, C. L., Ching, T. Y., Leigh, G., Cupples, L., Button, L., Marnane, V., Whitfield, J., Gunnourie, M., & Martin, L. (2018). Psychosocial development of 5-year-old children with hearing loss: Risks and protective factors. International Journal of Audiology, 57(sup2), S81–S92. 10.1080/14992027.2016.1211764 PMC5316508 27541363

[ref45] Woolfenden, S., Posada, N., Krchnakova, R., Crawford, J., Gilbert, J., Jursik, B., Sarkozy, V., Perkins, D., & Kemp, L. (2015). Equitable access to developmental surveillance and early intervention – Understanding the barriers for children from culturally and linguistically diverse (CALD) backgrounds. Health Expectations : An International Journal of Public Participation in Health Care and Health Policy, 18(6), 3286–3301. 10.1111/hex.12318 25510182 PMC5810693

[ref46] Wynaden, D., Chapman, R., Orb, A., McGowan, S., Zeeman, Z., & Yeak, S. (2005). Factors that influence Asian communities’ access to mental health care. International Journal of Mental Health Nursing, 14(2), 88–95. 10.1111/j.1440-0979.2005.00364.x 15896255

[ref47] Yoshinaga-Itano, C. (2003). From screening to early identification and intervention: Discovering predictors to successful outcomes for children with significant hearing loss. Journal of Deaf Studies and Deaf Education, 8(1), 11–30. 10.1093/deafed/8.1.11 15448044

[ref48] Yoshinaga-Itano, C. (2014). Principles and guidelines for early intervention after confirmation that a child is deaf or hard of hearing. Journal of Deaf Studies and Deaf Education, 19(2), 143–175. 10.1093/deafed/ent043 24131505

[ref49] Zhou, Q. (2016). Accessing disability services by people from culturally and linguistically diverse backgrounds in Australia. Disability and Rehabilitation, 38(9), 844–852. 10.3109/09638288.2015.1062925 26156203

